# The Reformulation of a Beef Patty Enriched with *n*-3 Fatty Acids and Vitamin D_3_ Influences Consumers’ Response under Different Information Scenarios

**DOI:** 10.3390/foods9040506

**Published:** 2020-04-17

**Authors:** Maria José Beriain, Inmaculada Gómez, Mercedes Sánchez, Kizkitza Insausti, María Victoria Sarriés, Francisco C Ibañez

**Affiliations:** 1 Research Institute for Innovation & Sustainable Development in Food Chain, Universidad Pública de Navarra, Campus de Arrosadía, 31006 Pamplona, Spain; mersan@unavarra.es (M.S.); kizkitza.insausti@unavarra.es (K.I.); vsarries@unavarra.es (M.V.S.); pi@unavarra.es (F.C.I.); 2 Departamento de Biotecnología y Ciencia de los Alimentos, Universidad de Burgos, 09001 Burgos, Spain; igbastida@ubu.es

**Keywords:** beef patty, consumer, information scenario, willingness to pay, purchase, acceptance

## Abstract

The objective of this study was to investigate the sensory acceptability and willingness to pay (WTP) for a beef patty elaborated with beef from a local breed that was enriched with nutritional ingredients (vegetable oil mixture and vitamin D_3_). The experiment was conducted under two information scenarios (blind; full: ingredients used to enrich the patties in *n*-3 PUFA and vitamin D_3_). An in-home use test was carried out by 180 consumers to study consumer liking of two low-fat beef patties (*C*: conventional, *M*: modified). There were no differences in color and odor for the raw patties (*p* > 0.05). The sensory parameters of the cooked patties were assessed as being similar (*p* > 0.05) regardless of the information scenario. The sensory parameters remained crucial criteria for product acceptance and repeat purchase. Consumers positively evaluated the *M* patty. The information provided to consumers on the composition of the product influenced the response of consumers. It was also observed that the higher the education level of the consumer, the higher their scores for *M* beef patties in the blind scenario. It is thus necessary to implement appropriate marketing strategies in order to highlight the nutritional properties of the modified patties, making them competitive ahead of conventional patties.

## 1. Introduction

Consumer demand in relation to food is increasingly tending towards food products that are safe, nutritious, elaborated through sustainable methods, and of good eating quality, showing the complexity of the actual consumer behavior [1,2]. In contrast to other food sectors, the meat industry has been relatively slow in reacting to some of these trends [3]. In Spain, for example, the decline in fresh meat consumption since 2008 has affected the whole meat sector [4]. Thus, understanding consumers’ perception of beef quality is of paramount importance for the industry in order to remain competitive in the market. At the consumer level, several studies have shown that the strongest quality attributes for beef are flavor, tenderness, juiciness, freshness, leanness, healthiness, and nutritional value as intrinsic quality cues, together with brands or labels as extrinsic quality cues [5,6,7]. Although before purchase, process-related characteristics, healthiness, appearance, and eating quality have similar weights in the formation of quality expectations, eating quality stands out as the most decisive criterion shaping quality experience, satisfaction or dissatisfaction, and future purchase [8].

Although innovation to improve processed meat products is promising, sensory characteristics remain the key factor shaping consumers’ preference and purchase decisions [9]. The growing consumer interest in healthier and safer meat products continues to shape the meat industry and its production [8,10,11], partly due to consumers’ fear resulting from consecutive waves of safety scares, adverse health effects, sustainability, and adulteration issues [12,13]. The growth of health conscientiousness and importance of healthy eating has been influencing consumers’ consumption, especially during the last few decades [14]. People pay more attention to their diet and tend to purchase products that provide health benefits, and this is particularly the case in developed countries due to the high number of elderly who are more concerned about their healthcare and the long working hours that often jeopardize the chance to meet dietary recommendations [15].

Red meat is considered to be an important part of a healthy balanced diet. Beef meat is a source of high value biological protein and important micronutrients, including vitamins B_6_ and B_12_, and *heme* iron [16]. However, over the last 10–15 years, these positive attributes have often been overshadowed due to some negative perceptions. The latest perception includes the fact that beef meat can contain variable amounts of saturated fat and potential carcinogens, the reason why red meat has been associated with cardiovascular diseases and cancer [3]. The putative relationship between dietary fat and incidence of non-communicable diseases has contributed to the development of specific guidelines from the World Health Organization (WHO) in relation to fat in the diet. It is recommended that total fat, saturated fatty acids (SFA), *n*-6 polyunsaturated fatty acids (PUFA), *n*-3 PUFA, and trans fatty acids should contribute < 15%–30%, < 10%, < 5%–8%, < 1%–2%, and < 1% of total energy intake, respectively. Reducing the intake of SFA and increasing the intake of *n*-3 PUFA is particularly encouraged. Among the *n*-3 PUFA, eicosapentaenoic (EPA) and docosahexaenoic (DHA) acids have been demonstrated as playing important roles in reducing the risk of cardiovascular diseases and may have an effect in reducing some cancers, obesity, and type-2 diabetes. Red meat, oily fish, and eggs are important sources of these *n*-3 PUFA for human health. However, the availability of long chain *n*-3 PUFA from fish appears to be limited due to their low consumption and to the concerns arising regarding the future sustainability of this protein source. This has resulted in increased attention being devoted to increasing these fatty acids (FAs) in other important food sources. Attention has also focused on the extent to which consumption of the precursor of the *n*-3 PUFA series, α-linolenic acid, can provide sufficient amounts of EPA and DHA through the *n*-3 PUFA elongation–desaturation pathway [17]. This process demands great efforts in research and development because fat contributes to sensory attributes such as tenderness, juiciness, and yield that are considered to be important by consumers [18].

Vitamin D deficiency is common worldwide, and therefore food fortification with this vitamin is a necessary strategy [19], as it is associated with many chronic illnesses [20]. Because the vitamin D content of meat is low, especially in low-fat meat products [21], increase in vitamin D content of meat products by technological methods is a challenge that needs to be overcome in the meat sector. Moreover, the increase in price, due to the application of these methods for the enrichment of meat with vitamin D, could modify the purchase intention of consumers. Thus, the study of the effect of nutritional claims on consumers’ willingness to pay (WTP) is necessary.

Different authors have evaluated the impact of food perception by changing their texture. A study conducted with the elderly, involving tasting different meat products, showed no significant impact of dental status on the food bolus formation. However, the age-related oral impairments are known to have an effect on food consumption. Therefore, there is a need to develop novel foods that meet specific texture for the elderly population [22,23]. Moreover, according to Escriba-Perez et al. [24], there is no general consumer behavior pattern for all meats, as each meat type has its own consumer profile. For example, young consumers in Spain seem to be the segment with the highest demand for minced beef carrying desirable labels such as “low fat”, “moderate fat”, and “local” [25]. In this sense, Hathwar et al. [26] considered health concerns and sociodemographic features among the most important factors influencing the changes in consumer demand for meat and meat products. Therefore, the marketing strategies have to be adapted to the heterogeneity of consumers’ preferences [24,25]. Thus, this study could benefit the meat sector, especially the beef sector, by providing information about a new product that consumers could identify as healthy, thereby helping and promoting beef consumption.

For all these reasons, the meat industry is interested in offering safer and healthier products by enhancing their nutritional composition, as well as convenient food products, achieved through modifying their textural properties [27].

In this context, the main goal of the present work was to investigate the sensory acceptability, purchase intention, and WTP of a beef patty elaborated with beef from a local breed, enriched with nutritional ingredients (vegetable oil mixture and vitamin D_3_). In addition to this, socioeconomic profiles of beef consumers that had similar purchase intentions and perceptions were defined. The study was conducted under two different situations (with and without information) to assess the effect of different factors on the individual response of consumers towards each product.

## 2. Materials and Methods

### 2.1. Design of the Study

**Figure 1:**
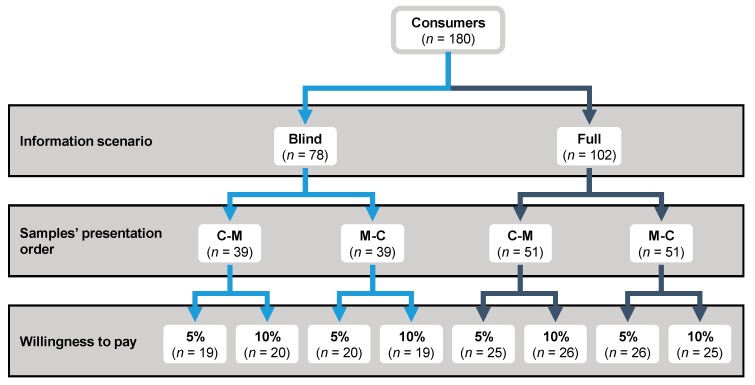
Experimental design used in the present study (*C*: conventional patty; *M*: modified patty). Information scenario: *Blind*: disclosure of details of the meat origin, *Full*: disclosure of details of the meat origin and the ingredients used to enrich the patties in *n-3* polyunsaturated fatty acids (PUFA) and vitamin D_3._

The consumer study was performed, aiming for representative conditions. An in-home use test of two low-fat beef patties (*C*: conventional; *M*: modified) was carried out because it is considered to be more realistic than laboratory testing and central location testing [28]. Study participants were assigned to one of the two scenarios: blind and full information disclosure, including details of the health properties of the products related to the content of *n*-3 PUFA and vitamin D_3_. In addition to this, consumers were asked to indicate if they were willing to pay an extra 5% or 10% for this type of product. With all these variables, eight different groups of consumers were studied (Figure 1).

### 2.2. Meat Products

Two formulations of ground beef patties (*C* and *M*) were elaborated. The elaboration procedure and the details of formulations were previously described by Gómez et al. [29]. Briefly, in the modified patty, 50% of backfat was replaced by 50% oil mixture (25% olive oil and 75% linseed oil) in water emulsion, and 8.3 μg vitamin D_3_/100 g product was added. These changes in the formulation led to increases in PUFA content in the cooked beef patties (*C*: 535.37, *M*: 953.50, mg FA/100 g product) and an important content of vitamin D_3_ in the cooked *M* patty (5.2 μg/100 g).

### 2.3. Screening of participants

The group of consumers who participated in this consumer test was recruited from the meat buyer population. The study was carried out in Northern Spain (Navarra). A total of 180 consumers, representative of population’s characteristics according to sex, age, and income level, was randomly selected to participate (according to the Spanish National Institute of Statistics). The participants were recruited among the regular purchasers of 15 local butcher shops, and participants were compensated with a gift for their participation when returning the questionnaire.

### 2.4. Design of Information Scenarios

The sensory analysis of consumers was evaluated using an adapted method of Beriain et al. [30]. Two different phases of consumer behavior (sensory assessment and purchase decision) at two information-availability scenarios were set up. In addition, the effect of socioeconomic characteristics on the valuations of the consumers was studied. All the consumers who participated were divided into two groups, each receiving different information. Thus, two experimental marketing scenarios were established: (a) at the blind scenario, only meat origin information was provided (meat from Protected Geographic Indication (PGI) “Ternera de Navarra”; (b) the full scenario included information on the meat origin and the ingredients used to enrich the patties in *n*-3 PUFA and vitamin D_3_. Consumer preferences for products and differences in intrinsic attributes and extrinsic attributes were analyzed (Table A1).

### 2.5. Procedure of the Consumer Study

**Figure 2:**
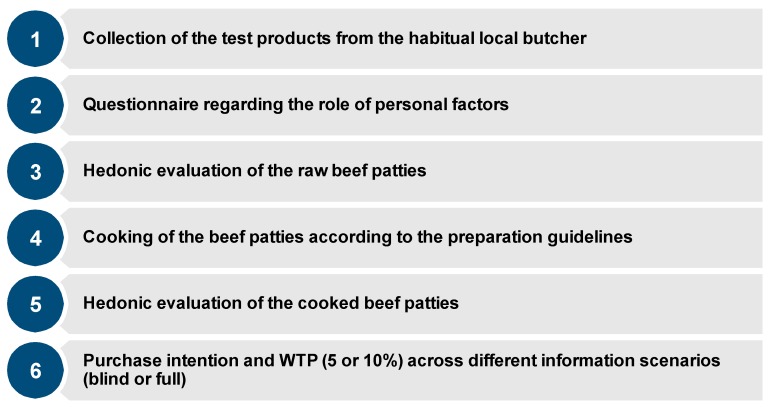
Procedure followed by consumers at the in-home use test.

The general procedure is shown in Figure 2. Participants collected their cooled test products with an enclosed questionnaire from their habitual local butcher. A bag was delivered to each participant with the following content: trial instructions, a consumer habit and preference questionnaire, two sensory evaluation scorecards (raw and cooked patties), and a questionnaire to study the purchase intention and WTP. The beef patties were marketed with a label according to the EU Regulation no. 1169/2011. The ingredients (oil mixture and vitamin D_3_ included) were written in the label. The *n*-3 fatty acid and vitamin D_3_ contents were included in the composition of the label. The nutritional claims were not in the label. The minimum amount to test was 50 g, which represents half of the amount of the provided test product (100 g). They were instructed to use the product on the same or following day according to preparation guidelines: “Add a small amount of sunflower oil to the frying pan and cook one patty when it is hot enough. Turn over the patty four times (1 minute per side). After approximately 4 min, the patty will be cooked and ready for tasting. Before each tasting, rinse your mouth by eating some bread and drinking some water. In order to avoid flavor cross-contamination, clean the frying pan before cooking the next sample or use a different pan”.

The test patties were only consumed by the participants, and they were not allowed to combine this with other meal components.

Questionnaires were designed to explore the role of personal factors in order to assess participants’ hedonic evaluations of raw and cooked patties and to study the purchase intention and WTP. Table A2 shows questions with different types of measurement scales in the responses about their consumption behavior, health concerns, WTP, and purchase intention.

### 2.6. Consumer Habits and Preferences Questionnaires

Some socioeconomic data and their meat purchase frequency were answered by the consumers. Secondly, consumers were asked to key quality cues using a guide for their choice of product. A Likert scale from 1 to 9, with 1 being “not at all important” and 9 “very important”, was used. 

### 2.7. Consumer Hedonic Evaluation

Consumer liking of beef patties was evaluated by the participants by in-home use test. Consumers tasted the samples in the order printed on the recording sheet according to Macfie et al. [31], in order to avoid sample order presentation, first-order, or carry-over effects.

Firstly, consumers evaluated the external aspect of the raw beef patties. Each consumer rated for odor, color, and appearance using a nine-point category scale from 1 “dislike extremely” to 9 “like extremely”) [32].

After cooking the beef patties according to the preparation guidelines, consumers evaluated the cooked beef patties. Each consumer rated for aroma, juiciness, tenderness, flavor, and overall acceptability using the nine-point category scale previously explained.

### 2.8. Purchase Intention and WTP

Products were evaluated across different information settings. The purchase intention was evaluated using a scale of 10 points following the method used by others [32,33,34,35,36]. One of the main goals of the present work was to decide the effect of different quality attributes to measure WTP for the patties. The authors’ proposal was the contingent valuation method [37] adapted from Clark et al. [38] and Napolitano et al. [39]. In this way, WTP was calculated with a simulation of a hypothetical market, with which the consumer assumed the supply and demand shown by the subject [33,34]. Consumers were asked to indicate if they were willing to pay an extra 5% or 10% for this type of product. If they were not willing to pay a premium, they were requested to support their answer by providing reasoned information (similar quality, the taste, or the inappropriate visual aspect). If they were willing to pay an extra 5% or 10%, after that, they were asked to state the highest amount that they would be willing to pay.

### 2.9. Statistical Analysis

The methodological techniques used in this study were the analysis of variance (ANOVA), logistic regression models, and principal components factorial analysis. Furthermore, doubly censored Tobit models, and Heckman models were always used.

Analysis of variance was used to assess the influence of the differences between conventional (*C*) and modified (*M*) patties and across experimental treatments, that is, the impact of the degree of information provided to survey participants on sensory ratings. All the sensory attributes were studied by applying the same model. Formulation patty and information level were considered as fixed effects using the following model:

*Y_ij_* = *μ* + *X_i_* + *S_j_* + *ε_ijk_* (1)

where *Y* = the study variable, *μ* = the least squares mean, *X_i_* = the type of patty (*i* = 1 if *C* patty, *i* = 2 if *M* patty), *S_j_* = the information level or experimental treatment, and *ε_ijk_* = the random term.

Due to the fact that panelist factor is not relevant, it was not considered in this study.

The ANOVA procedure was also used to analyze differences in purchase intention of the evaluated products across the different information scenarios.

As it was also the case in previous research, the product attributes were categorized as intrinsic or extrinsic [5]. A hierarchical Likert scale of 1–9, where 1 represents the minimum level and 9 shows the maximum grade of importance, was used to allow the survey participants to evaluate these cues. Scale reliability was tested using of confirmatory factor analysis. Table A1 shows the scores, which fell within the acceptable range.

The first factor, identified as additional components, included vitamins, *n*-3 PUFA, natural antioxidants, protein content, and health information [35]. The second factor, identified as intrinsic attributes, included flavor, freshness, tenderness, color, and additional aspects linked to ”expiration date information”. The third factor, identified as extrinsic attributes, included label, packaging, ready to cook, and healthy food guarantee. The fourth factor was associated with geographical origin relevance, and the last factor with price importance. On the other hand, another factor analysis was employed to decide the main aspects in consumer attitude towards innovation and food products (Table A2). Therefore, the first factor included interest in new products, the second one the relevance of health food information [35], and the last one represented the lower interest in new food.

The extent to which intent to purchase *C* and *M* patties, which were influenced by the already-mentioned sociodemographic characteristics of the purchaser, pre-purchase quality cues, were estimated by doubly censored Tobit models. Then, the attitude towards new food and overall acceptability of the patty tested under each information scenario were determined. Stata ver. 16 software was used (StataCorp LLC, Texas, USA).

To determine whether WTP was a premium, the Heckman model, with a two-stage decision, was estimated. The first decision studied the factors that influenced willingness to pay or not, and the second decision analyzed the factors that affected the final amount of WTP. In addition, whether the two decisions were simultaneous or sequential was able to be tested.

Statistical analyses were carried out using the package IBM SPSS Statistics version 24 (IBM Corp., New York, NY, USA).

## 3. Results

### 3.1. General Description of the Consumer Sample by Information Scenario

Table 1 provides the characteristics of the consumer sample by information settings (blind vs. full). The higher proportion of women than men in the consumer sample was because the main household food purchaser still tends to be a woman in Spain [36]. For the remainder, the sample was representative of the reference population, the survey region being representative of Spain as a whole with respect to the market that concerns the present study. In general, over 80% of the consumers of this study consumed beef once a week or more, whereas the other consumers reported occasional consumption. Beef meat consumption in 2014 throughout the region was over the Spanish national average (Spain—5.88 kg beef per capita, and Navarra—6.41 kg per capita) [4]. Males over 50 years of age belonging to the low, lower-middle, and middle-classes and with an elementary education, reported higher weekly beef consumption frequency (more than once a week) than upper-class females with a higher education under 50 years of age (once a week). These results were in agreement with the standard consumption patterns of this geographical location.

The sociodemographic profiles of the two sub-samples showed no statistically significant differences for the studied characteristics, except for income level.

### 3.2. Effect of the Information Scenario on the Sensory Analysis

Table 2 shows the least square means (LSM), standard deviation (SD), and *p*-values obtained after applying the analysis of variance to assess the influence of differences between *C* and *M* patty (conventional vs. modified with olive and linseed oils mixture plus vitamin D_3_) and the impact of the degree of information provided to the consumer survey participants (blind scenario vs. full scenario) on sensory ratings evaluated in raw patties before cooking—color, odor, and overall acceptability.

Consumers detected slight differences for color (*p* = 0.089) and significant differences for overall acceptability (*p* = 0.046). In this sense, conventional patties reached a higher color score (5.82 vs. 5.52) and overall acceptability score (5.93 vs. 5.58) than patties with modified formulation. No statistically significant differences (*p* > 0.05) were observed for odor.

Table 3 displays the LSM, SD, and *p*-values obtained after applying the analysis of variance to assess the influence of differences between *C* and *M* patty (conventional vs. modified with olive and linseed oil mixture plus vitamin D_3_) and the impact of the degree of information provided (blind scenario vs. full scenario) to survey participants on sensory ratings evaluated in cooked patties—flavor, tenderness, aroma, juiciness, and overall acceptability. From these results, it can be stated that consumers did not detect any statistically significant difference due to either the composition of the patties or the different level of information in any of the sensory attributes evaluated (*p* > 0.05). In addition to this, it is shown that the cooked patties enriched with a vegetable oil mixture and vitamin D_3_ obtained the same values in the sensory attributes as those obtained in the conventional patty. Moreover, in relation to the level of information provided to consumers, the sensory results of the cooked patties reached the same scores regardless of whether or not consumers received information prior to performing the sensory test.

### 3.3. Effects of Market Factors on Sensory Quality and Purchase Intention and WTP

The effect of sociodemographic factors (age, gender, social status, educational level, and employment status) and the patties’ composition (*C* or *M*) on consumer scores for sensory descriptors of raw and cooked patties were studied (Table 4). In any case, neither in the raw patties nor in the cooked patties were significant interactions found between the patty composition and each of the sociodemographic factors analyzed. In addition, the effect of the patties’ composition was not significant for any of the sensory parameters studied. However, there were significant differences in these attributes that were dependent on age, social status, educational level, and employment status of the participants in the study. In general, the highest scores were obtained for flavor and overall acceptability attributes in cooked patties. The consumers that rated the higher scores of patties, both raw and cooked, were consumers over 65 years old, with a medium-high social status, a medium education level, and were retired. The factors that most influenced sensory assessments were the employment situation and age.

Table 5 illustrates Tobit models of the influence of sociodemographic factors, consumption frequency, pre-purchase quality cues, and acceptance on hedonic attribute ratings by information scenario. The gender effect emerged in the fact that women tended to rate the modified patties higher than the conventional beef patty. Higher education was associated with higher scores for *M* beef patties in the blind scenario. The information group was influenced by the gender and extrinsic cues, and thus men with more information (0.67) and those that appreciated the extrinsic cues (0.38) in the buying process showed more willingness to buy the meat product (Table 5). On the other hand, the highest levels of product acceptability were associated with higher purchase interest for both consumer information scenarios (1.22 and 0.79 (blind scenario) and 1.14 and 1.10 (full information)) (Table 5).

**Figure 3:**
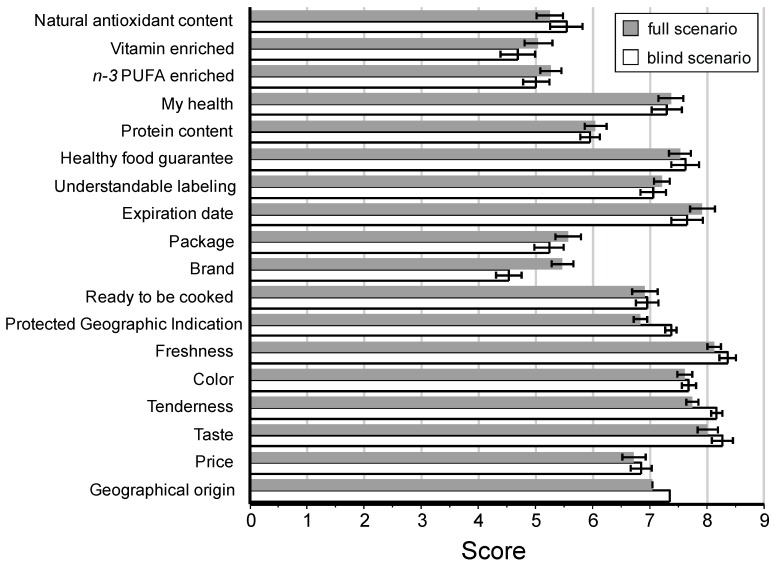
Evaluation of the quality cues that guide consumers to their choice of product. Scale from 1 to 9, with 1 being “not at all important” and 9 “very important”. Error bars denote Standard Error of the Mean (SEM). Information scenario: *Blind*: disclosure of details of the meat origin, *Full*: disclosure of details of the meat origin and the ingredients used to enrich the patties in *n*-3 PUFA and vitamin D_3_.

Figure 3 shows the evaluation of the cues that guide consumers to their choice of product at the time of the purchase by information scenario. Following the results obtained by the analysis of the main aspects evaluated by the consumers, similar values were observed between both scenarios (blind vs. full). According to the used scale (from 1 to 9, with 1 being “not at all important” and 9 “very important”), a high score meant that an attribute was considered to be important for consumers. Thus, the most important aspects for consumers were freshness, taste, tenderness, expiration date, color, and healthy food guarantee.

**Figure 4:**
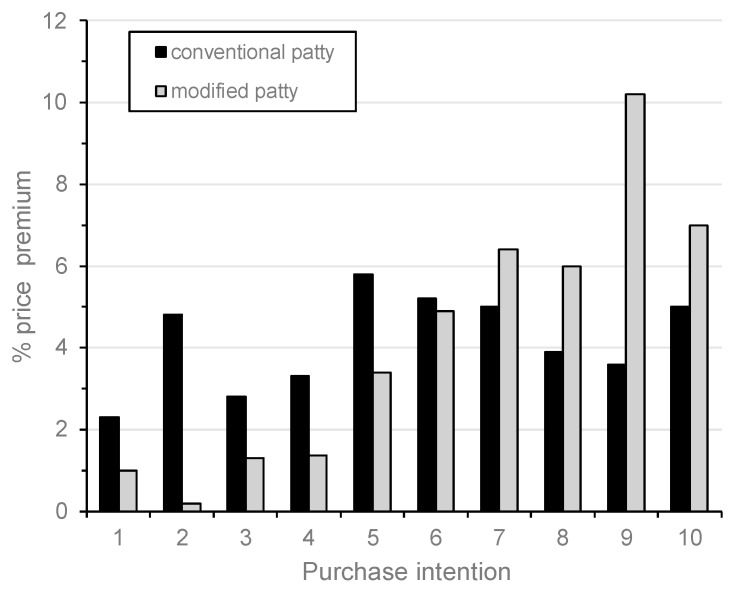
Relationship between willingness to pay (WTP) and purchase intention. Purchase intention (scale 1–10) where 1 = definitely would not pay, 10 = definitely would buy. Percentage price premium for the beef patties enriched with *n*-3 PUFA and vitamin D_3_.

Moreover, it is possible to observe differences in the price premium between both scenarios (blind vs. full). Figure 4 shows the relationship between WTP and purchase intention for the conventional patty and the modified patty, respectively. As expected, the consumers most interested in purchasing the modified patty showed more WTP for this product.

**Figure 5:**
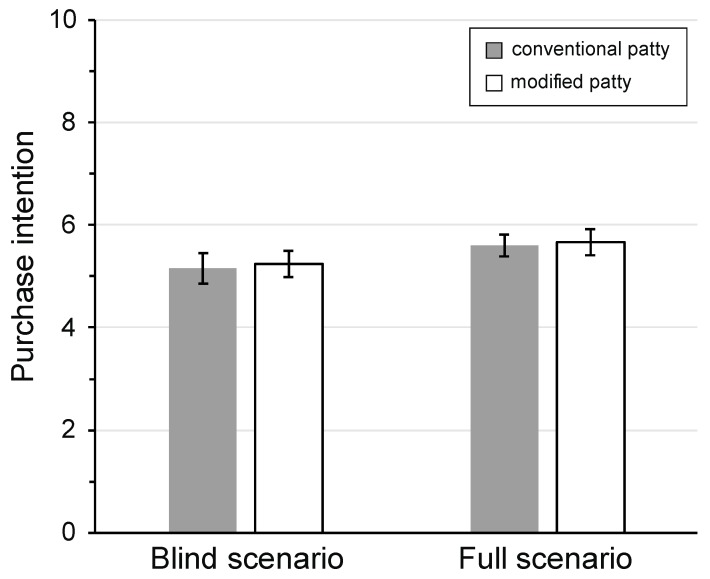
Purchase intention by composition and information scenario. One-way test for differences in purchase intention of the two beef patties. Purchase intention (scale 1—10) where 1 = definitely would not buy, 10 = definitely would buy. Error bars denote **S**tandard **E**rror of the **M**ean (SEM). There were no significant differences between patties in both information scenarios. Information scenario: *Blind*: disclosure of details of the meat origin, *Full*: disclosure of details of the meat origin and the ingredients used to enrich the patties in *n*-3 PUFA and vitamin D_3_.

In general, a greater impact on the *M* patty than the *C* patty across all treatment groups was shown due to various socioeconomic factors and purchase cues. Studied models showed that as purchasers’ access to information about the products increased, a larger number of factors impacted their sensory ratings. In sum, the mean scores for the *C* patty were lower than for the *M* patty in both information settings. This result had relevance for product composition and marketing, as these factors showed the important effect of marketing decisions, particularly those related to the information to disclose to prospective customers in relationship to product success. Additionally, in terms of purchase intention, the differences between information scenarios had no statistical differences (*p* = 0.13) (Figure 5). Consumers under the high-information scenario had a purchase intention higher than 5.5 compared to those who had the blind scenario, as they turned out to be less than 5.3.

Table 6 shows that the hedonic rating had a major effect on purchase intention for both patties under all information settings. Likewise, the Heckman models allowed us to find a positive association between higher purchase intention and liking for the products’ sensory attributes, indicating the degree of the coherence in the scoring. Second, an increasing effect on the purchase intention towards the modified patty was found by presenting biocompounds with nutritional properties. In this respect, consumers tended to use nutritional claims as quality keys prior to purchase.

Lastly, the sociodemographic factors with the highest impact on purchase intention were income and education, with some variation across information settings and between the two products.

## 4. Discussion

Despite efforts to investigate the technical feasibility of achieving nutritional attributes in meat products, few studies have addressed consumer perceptions regarding these products.

Although this market is promising, it is important to understand consumers’ perceptions and attitudes towards new products to achieve appropriate product positioning [37,38]. To better understand consumer’s choice, some authors have endorsed the use of indirect research methods. The evaluation of individuals’ behavior regarding food may provide insight into factors that influence consumer choices [39]. The few differences found by consumers between both types of beef patties in this work could be related to the fact that an in-home use test was used, and when satisfaction with preparation is taken into account, the home use test provides more understandable information about consumers’ assessments [40].

Meanwhile, sensory characteristics such as taste remain crucial criteria for product acceptance, trial, and repeat purchase [41,42]. Our results corroborate this statement because the taste, together with the freshness of the products, were the main criteria of choice for both scenarios (Figure 3). The concept of these new processed meat products has been favorably evaluated by stakeholders and consumers [9,43,44].

In the present study, it was people over 65 with a higher level of education and economic level who best valued the characteristics of the modified patties. In a previous work [30,45], authors reported that the most influential socioeconomic variables were consumer gender and age. In addition, quality-conscious consumers showing higher WTP for extra quality also showed previous higher purchase intention.

In previous studies, authors analyzed the effect of available information and consumer characteristics on purchase intention and WTP for a claimed nutritional property, finding that availability of information had a positive effect on identification, price, and nutritional benefit [30,45]. The results obtained in the present study corroborates these findings.

Concerning nutritional components, *n*-3 PUFA, and vitamin D_3_ were preferred by consumers with a high purchase intention (Figure 5). Modified reformulations improved the perceived nutritious perception of processed meats. Thus, healthy component enrichment can improve the health image of processed meat [46].

It also appears that psychometric variables in association with sociodemographic, cognitive, and attitudinal factors play a role in consumers’ purchase decisions [47]. Women were more interested than men in having information about nutritive products before buying them (women = 7.05; men = 6.40; *p* < 0.036) and they were also the ones who buy more enriched foods compared to men (women = 4.85; men = 4.07; *p* < 0.056).

Sociodemographic factors may influence the health perception and consumption frequency of processed meats. For instance, in the present work, as in previous works, it has been found that women are more critical and consume less processed meat than men [48,49,50,51]. It should be noted that when origin information was entered, higher-educated purchasers abandoned their preference for the *C* patty, as their scores under the high-information settings were higher for the *M* patty. Although the highest scores were for the modified patties, this fact can be associated with the group of consumers whose source of income was the highest and in the blind scenario. However, these latter associations did not occur in the scenario with information, as there may have been an effect related to the repetition of information when testing several products.

Modified patties could have a high level of acceptability in elderly people with difficulties in chewing because they have a higher tenderness and contain amounts of *n*-3 PUFA and vitamin D_3_ that allow coverage of the nutritional requirements of both nutrients. Similar conclusions came from previous studies [52], which reported that university consumers may accept these healthier substitutes for traditional full-fat beef patties. Consumer acceptance of healthier patty substitutes should be further investigated in primary and secondary schools as well.

The analysis of consumer behavior towards the two different patties in terms of health has shown interesting results, especially with relation to product attributes evaluated, sociodemographic consumer aspects, and WTP and purchase intention. These results showed the complexity of the food consumer behavior and the effect of the sensorial, context, cultural, sociodemographic, psychological, and product aspects [1,2]. Furthermore, a higher level of education, less income, and less consumer frequency led to a higher sensory evaluation of the modified patties. Moreover, people who were more interested in these modified patties showed higher levels of WTP. For that reason, a good level of acceptability is required to create a positive market response, and an increase in consumers’ purchase intention of beef patties is the main finding that emerged from the estimation of these models. This shows the interaction between the production of nutritional meat products and marketing in the agro-food sector.

## 5. Conclusions

The sensory characteristics remain crucial criteria for product acceptance, trial, and repeated purchase. The concept of this modified patty has been favorably evaluated by consumers. With proper marketing, nutritious alternatives to the conventional, full-fat patty could become competitive choices.

Nutritional component enrichment can improve the image of processed meat; however, the type of ingredient should be carefully selected to maximize the likelihood of consumer purchase. Only when the nutritional information is relevant and understandable for consumers is the perception of meat enriched with healthier ingredients improved and the WTP increased.

In order to successfully market these new meat products, consumer profile and product attributes must be considered. This could make the marketing strategies more difficult, but at this moment, it is necessary to have an adequate development in the saturated agri-food market. In addition to sensorial analysis, different information scenarios and some aspects of consumer behavior could help to define the marketing tools. Thus, the information presented in this paper could be of great practical importance for the meat sector.

Taking into account the interaction that consumer health concerns might have with other consumer preferences, future research should consider interaction effects between health-related attributes and other desirable attributes (e.g., organic, local, sustainable) in order to avoid biased and misleading results.

## Figures and Tables

**Table 1:** General description of the consumer sample by information scenario.

	Variable	Definition	Total	Blind Scenario (*n* = 78)	Full Scenario (*n* = 102)	Statistical Value	*p*-Value
Sociodemographic characteristics	Gender	Male	39.0%	40.0%	60.0%	0.518^A^	0.470
		Female	61.0%	45.5%	54.5%		
	Age (years)		43.6	44.4	43.0	0.518^B^	0.480
	Education level	Elementary	15.0%	55.6%	44.4%	1.963^A^	0.380
		Secondary	34.4%	40.3%	59.7%		
		Higher	50.6%	41.8%	58.2%		
	Income level	Modest	10.0%	55.6%	44.4%	6.345^A^	0.040
		Medium	76.7%	45.7%	54.3%		
		High	13.3%	6.4%	18.6%		
Meat consumption frequency (per week)			1.70	1.83	1.61	2.676^B^	0.104

^A^ Pearson’s χ^2^; ^B^ Snedecor’s F. Information scenario: *Blind*: disclosure of details of the meat origin, *Full*: disclosure of details of the meat origin and the ingredients used to enrich the patties in *n-3* PUFA and vitamin D_3_.

**Table 2:** Least square means (LSM), standard deviation (SD), and *p*-values of consumer scores for sensory descriptors of raw patties (conventional, and modified with olive and linseed oil mixture and vitamin D_3_) on the different information levels.

	Information Scenario	Color	Odor	Appearance
Conventional patty	Blind scenario	5.6 (1.7)	5.3 (1.2)	5.8 (1.7)
	Full scenario	5.6 (1.5)	5.6 (1.3)	6.0 (1.4)
	*p*-value	0.166	0.100	0.263
	LSM (SD)	5.8 (1.6)	5.5 (1.3)	5.9 (1.6)
Modified patty	Blind scenario	5.6 (1.3)	5.4 (1.6)	5.5 (1.7)
	Full scenario	5.5 (1.5)	5.5 (1.4)	5.6 (1.5)
	*p*-value	0.745	0.716	0.673
	LSM (SD)	5.5 (1.5)	5.6 (1.3)	5.6 (1.6)
*p*-value	Composition	0.089	0.391	0.046
	Information level	0.437	0.158	0.626

Information scenario: *Blind*: disclosure of details of the meat origin, *Full*: disclosure of details of the meat origin and the ingredients used to enrich the patties in *n*-3 PUFA and vitamin D_3._ Nine-point scale where: 1 = dislike extremely, 2 = dislike very much, 3 = dislike moderately, 4 = dislike slightly, 5 = neither like nor dislike, 6 = like slightly, 7 = like moderately, 8 = like very much, 9 = like extremely.

**Table 3:** Least square means (LSM), standard deviation (SD), and *p*-values of consumer scores for sensory descriptors of the cooked patties (conventional, and modified with olive and linseed oil mixture and vitamin D_3_) on the different information levels.

	Information Scenario	Aroma	Juiciness	Tenderness	Flavor	Overall Acceptability
Conventional patty	Blind scenario	5.7 (1.5)	5.7 (1.5)	5.7 (1.4)	6.0 (1.7)	5.9 (1.6)
	Full scenario	6.2 (1.4)	5.7 (1.7)	5.9 (1.7)	6.2 (1.7)	5.6 (1.3)
	*p*-value	0.020	0.892	0.502	0.155	0.229
	LSM (SD)	6.0 (1.5)	5.7 (1.6)	5.8 (1.6)	5.2 (1.7)	6.1 (1.7)
Modified patty	Blind scenario	6.1 (1.3)	5.9 (1.4)	6.0 (1.5)	6.1 (1.5)	6.1 (1.5)
	Full scenario	6.0 (1.5)	6.0 (1.7)	6.0 (1.7)	6.3 (1.6)	6.2 (1.6)
	*p*-value	0.633	0.543	0.718	0.398	0.474
	LSM (SD)	6.0 (1.4)	6.0 (1.6)	6.0 (1.6)	6.2 (1.6)	6.1 (1.5)
*p*-value	Composition	0.418	0.146	0.256	0.975	0.464
	Information level	0.176	0.602	0.466	0.105	0.171

Information scenario: *Blind*: disclosure of details of the meat origin, *Full*: disclosure of details of the meat origin and the ingredients used to enrich the patties in *n*-3 PUFA and vitamin D_3_. Nine-point scale where: 1 = dislike extremely, 2 = dislike very much, 3 = dislike moderately, 4 = dislike slightly, 5 = neither like nor dislike, 6 = like slightly, 7 = like moderately, 8 = like very much, 9 = like extremely.

**Table 4:** Least square means (LSM), standard deviation (SD), and *p*-values of consumer scores for sensory descriptors of the raw and cooked patties (conventional, and modified with olive and linseed oil mixture and vitamin D_3_) according to sociodemographic factors.

		Raw Patty			Cooked Patty					
Variable	Definition	Odor	Color	Appearance	Aroma	Juiciness	Tenderness	Flavor	Overall Acceptability	Purchase Intention
Age	20–34	5.5 (1.4)	5.5 (1.5)	5.5 (1.5)	6.1 (1.4)	5.9 (1.5)	5.9 (1.5)	6.1 (1.6)	6.2 (1.5)	5.4 (2.2)
	35–50	5.4 (1.2)	5.6 (1.5)	5.8 (1.5)	5.9 (1.4)	5.9 (1.5)	5.9 (1.5)	6.3 (1.5)	6.1 (1.5)	5.7 (2.5)
	50–65	5.4 (1.3)	5.6 (1.5)	5.8 (1.7)	5.6 (1.4)	5.5 (1.8)	5.5 (1.7)	5.9 (1.8)	5.8 (1.8)	4.9 (2.5)
	>65	6.6 (1.8)	6.9 (1.9)	6.8 (1.9)	7.1 (1.8)	6.5 (1.3)	7.4 (1.4)	7.2 (1.7)	7.2 (1.7)	6.3 (1.5)
	*p*-value	0.001	0.003	0.008	0.000	0.04	0.000	0.019	0.002	0.028
Economic status	Medium-low	5.5 (1.5)	5.5 (1.7)	5.7 (1.9)	5.8 (1.5)	5.5 (1.7)	6.1 (1.6)	6.2 (1.7)	5.9 (1.8)	4.7 (2.4)
	Medium	5.4 (1.3)	5.6 (1.6)	5.7 (1.6)	5.9 (1.4)	5.8 (1.6)	5.7 (1.6)	6.1 (1.7)	6.1 (1.6)	5.3 (2.4)
	Medium-high	5.9 (1.4)	6.1 (1.5)	6.0 (1.4)	6.5 (1.6)	6.2 (1.4)	6.7 (1.5)	6.8 (1.6)	6.8 (1.6)	6.7 (1.7)
	*p*-value	0.096	0.192	0.534	0.027	0.080	0.000	0.016	0.004	0.000
Education level	Elementary	5.2 (1.4)	5.2 (1.9)	5.8 (2.1)	5.6 (1.6)	5.46 (1.8)	5.8 (1.8)	6.0 (1.7)	5.9 (1.8)	5.0 (2.7)
	Secondary	5.7 (1.3)	5.9 (1.4)	5.9 (1.4)	5.9 (1.5)	5.9 (1.6)	5.9 (1.6)	6.3 (1.7)	6.2 (1.6)	5.5 (2.4)
	Higher	5.5 (1.3)	5.7 (1.5)	5.6 (1.5)	6.2 (1.4)	5.9 (1.5)	5.9 (1.5)	6.2 (1.6)	6.1 (1.6)	5.5 (2.3)
	*p*-value	0.118	0.042	0.274	0.016	0.158	0.889	0.442	0.578	0.343
Employment status	Student	5.3 (1.3)	5.8 (1.7)	5.8 (1.7)	5.9 (1.7)	5.9 (1.8)	5.7 (1.8)	6.21 (2.1)	6.3 (1.8)	6.4 (2.2)
	Employee	5.4 (1.3)	5.6 (1.4)	5.8 (1.5)	5.9 (1.4)	5.7 (1.6)	5.8 (1.6)	6.2 (1.5)	6.1 (1.6)	5.3 (2.4)
	Entrepreneur	5.0 (1.2)	4.9 (1.6)	4.7 (1.7)	5.5 (1.4)	5.4 (1.7)	5.5 (1.7)	5.76 (1.9)	5.6 (1.8)	4.3 (2.6)
	Retiree	6.4 (1.6)	6.9 (1.6)	6.6 (1.7)	6.8 (1.7)	6.5 (1.3)	6.9 (1.5)	7.2 (1.5)	7.1 (1.5)	6.7 (1.4)
	Homemaker	6.1 (1.3)	6.3 (1.4)	6.1 (1.4)	6.0 (1.6)	6.3 (0.8)	6.5 (1.4)	6.7 (1.9)	6.6 (1.6)	6.7 (1.9)
	Unemployed	5.9 (1.2)	5.9 (1.6)	5.9 (1.3)	6.4 (1.2)	6.2 (1.3)	6.2 (1.3)	6.2 (1.5)	6.1 (1.1)	5.2 (2.1)
	*p*-value	0.000	0.000	0.000	0.004	0.034	0.005	0.027	0.010	0.000

Nine-point scale where: 1 = dislike extremely, 2 = dislike very much, 3 = dislike moderately, 4 = dislike slightly, 5 = neither like nor dislike, 6 = like slightly, 7 = like moderately, 8 = like very much, 9 = like extremely.

**Table 5:** Tobit models of the influence of sociodemographic factors, consumption frequency, pre-purchase quality cues, and acceptance on willingness to pay (by information scenario).

	Blind Scenario		Full Scenario	
	Conventional Patty (*n* = 78)	Modified Patty (*n* = 102)	Conventional Patty (*n* = 78)	Modified Patty (*n* = 102)
Intercept	−2.13	−1.96	−3.45	−4.22
Gender	−0.12	0.29	−0.29	0.67 *
Age	−0.02	−0.02	0.001	−0.02
Education level	−0.16	0.69 **	−0.29	0.44
Household income	0.26	0.72	1.47 **	0.98
Consumption frequency	0.25	0.29	−0.13	0.34
Urban habitat	0.55	−0.29	0.41	−0.18
Additional components	−0.25	0.17	0.15	−0.03
Intrinsic cues	−0.26	−0.23	−0.09	0.21
Extrinsic cues	−0.07	0.23	0.19	0.38 *
Geographical origin aspects	−0.68 ***	0.30 *	−0.71 ***	0.26
Price	0.45 **	0.10	0.10	−0.32
Interest in new food products	0.29	0.12	0.28	0.21
Interest in health information	0.13	0.34	−0.07	0.17
Less interest in new food products	0.19	−0.14	0.11	0.21
Acceptability	1.22 ***	0.79 ***	1.14 ***	1.10 ***
Log-likelihood	−136.41	−191.21	−135.14	−195.95

Significance level: * *p* < 0.05; ** *p* < 0.01; *** *p* < 0.001; not significant: *p* >0.05

**Table 6:** Heckman models of the influence of sociodemographic factors, consumption frequency, pre-purchase quality cues, overall general acceptability, and purchase intention on willingness to pay for patties by information scenario.

	Blind Scenario		Full Scenario	
	Equation 1	Equation 2	Equation 1	Equation 2
Intercept	1.95	−4.54	2.41 **	−2.47
Gender	−0.14	0.27	−0.07	−0.15
Age	−0.012 *	0.02	−0.009 *	0.009
Education	0.29	0.76 **	−0.26 **	0.14
Income	−0.31	−0.22	−0.45 *	−1.22
Urban habitat	0.19	−0.28	0.10	0.31
Consumption frequency	−0.18 ***	−0.20	−0.13 *	0.07
Additional components	0.18 **	0.11	−0.18 *	−0.006
Intrinsic cues	−0.02	−0.25	0.09	0.22
Extrinsic cues	−0.11	−0.29	−0.13	−0.17
Geographical origin aspects	−0.04	0.10	−0.05	−0.30 *
Price	0.03	0.26	−0.10	−0.04
Interest in new food	0.11 *	0.15	0.03	0.10
Interest in health information	0.26 ***	0.03	0.10	0.09
Less interest in new food products	−0.04	0.12	0.03	−0.08
Acceptability	−0.25 ***	0.06	−0.04	0.33 **
Purchase intention	0.15	0.42 ***	0.08	0.37 ***
Wald’s χ^2^	68.96 ***		23.09 *	
Lambda (sig)		0.1737		0.034

Significance level: * *p* < 0.05; ** *p* < 0.01; *** *p* < 0.001; not significant: *p* > 0.05. Information scenario: *Blind*: disclosure of details of the meat origin, *Full*: disclosure of details of the meat origin and the ingredients used to enrich the patties in *n*-3 PUFA and vitamin D_3_. Equation 1: Logistic regression to explain the willingness to pay for patties (1 willingness to buy and 0 in other case); Equation 2: Linear regression to explain the final amount of willingness to pay for patties.

## Appendix A

**Table A1:** Factor analysis of quality cues relevant to the purchase of beef.

Variable	Mean (SD)	Correlation Factor	α
*Additional components (% variance: 35.8%)*			0.882
Vitamin-enriched	4.89 (2.40)	0.853	
Omega *n-3* enriched	5.16 (2.57)	0.852	
Natural antioxidants	5.38 (2.49)	0.825	
Protein content	6.01 (2.25)	0.665	
Health importance	7.34 (1.95)	0.585	
Label information	7.14 (2.04)	0.491	
*Intrinsic cues (% variance: 11.21%)*			0.763
Flavor	8.12 (1.01)	0.793	
Freshness	8.23 (1.06)	0.783	
Tenderness	7.92 (1.28)	0.758	
Color	7.64 (1.23)	0.676	
Expiration date information	7.81 (1.69)	0.454	
*Extrinsic cues (% variance: 7.21%)*			0.723
Label	5.06 (2.32)	0.778	
Packaging	5.43 (2.28)	0.724	
Ready to cook	6.93 (1.93)	0.536	
Healthy food guarantee	7.57 (1.73)	0.464	
*Origin relevance (% variance: 6.6%)*			0.706
Denomination of origin	7.07 (2.06)	0.796	
Geographical origin	7.18 (1.88)	0.783	
*Price relevance (% variance: 5.6%)*			
Price	6.77 (1.75)	0.854	

α: Cronbach’s coefficient alpha higher than 0.70. SD: standard deviation.

**Table A2:** Factor analysis of attitude towards innovation and food products.

Variable	Mean (SD)	Correlation Factor	α
*Interest in new products (% variance: 34.46%)*			0.859
I am a first buyer of new food products	3.13 (2.03)	0.910	
I buy new food immediately	3.22 (2.06)	0.898	
I am very interested in new food	4.55 (2.11)	0.765	
*Interest in healthy food information (% variance: 18.54%)*			0.732
I buy healthy food	7.02 (1.79)	0.801	
I have learnt about food information previously	6.80 (2.05)	0.765	
I read food label information	5.81 (2.37)	0.747	
I am interested in new beef products	6.66 (2.06)	0.610	
*Less interest in new food products (% variance: 12.21%)*			
I am not interested in new food	4.18 (2.14)	0.757	
I previously taste before buying new food	4.32 (2.56)	-0.609	
I buy food enriched with healthy components	4.54 (2.65)	0.501	

α: Cronbach’s coefficient alpha higher than 0.70. SD: standard deviation.
